# Fisetin, a dietary flavonoid, augments the anti-invasive and anti-metastatic potential of sorafenib in melanoma

**DOI:** 10.18632/oncotarget.6237

**Published:** 2015-10-26

**Authors:** Harish C. Pal, Ariana C. Diamond, Leah R. Strickland, John C. Kappes, Santosh K. Katiyar, Craig A. Elmets, Mohammad Athar, Farrukh Afaq

**Affiliations:** ^1^ Department of Dermatology, University of Alabama at Birmingham, Birmingham, Alabama, USA; ^2^ Department of Medicine, University of Alabama at Birmingham, Birmingham, Alabama, USA; ^3^ Comprehensive Cancer Center, University of Alabama at Birmingham, Birmingham, Alabama, USA

**Keywords:** melanoma, fisetin, sorafenib, EMT, invasion

## Abstract

Melanoma is the most aggressive and deadly form of cutaneous neoplasm due to its propensity to metastasize. Oncogenic BRAF drives sustained activation of the BRAF/MEK/ERK (MAPK) pathway and cooperates with PI3K/AKT/mTOR (PI3K) signaling to induce epithelial to mesenchymal transition (EMT), leading to cell invasion and metastasis. Therefore, targeting these pathways is a promising preventive/therapeutic strategy. We have shown that fisetin, a flavonoid, reduces human melanoma cell invasion by inhibiting EMT. In addition, fisetin inhibited melanoma cell proliferation and tumor growth by downregulating the PI3K pathway. In this investigation, we aimed to determine whether fisetin can potentiate the anti-invasive and anti-metastatic effects of sorafenib in BRAF-mutated melanoma. We found that combination treatment (fisetin + sorafenib) more effectively reduced the migration and invasion of BRAF-mutated melanoma cells both *in vitro* and in raft cultures compared to individual agents. Combination treatment also effectively inhibited EMT as observed by a decrease in N-cadherin, vimentin and fibronectin and an increase in E-cadherin both *in vitro* and in xenograft tumors. Furthermore, combination therapy effectively inhibited Snail1, Twist1, Slug and ZEB1 protein expression compared to monotherapy. The expression of MMP-2 and MMP-9 in xenograft tumors was further reduced in combination treatment compared to individual agents. Bioluminescent imaging of athymic mice, intravenously injected with stably transfected CMV-luciferase-ires-puromycin. T2A.EGFP-tagged A375 melanoma cells, demonstrated fewer lung metastases following combination treatment versus monotherapy. Our findings demonstrate that fisetin potentiates the anti-invasive and anti-metastatic effects of sorafenib. Our data suggest that fisetin may be a worthy adjuvant chemotherapy for the management of melanoma.

## INTRODUCTION

Over 90% of cancer-related deaths are attributable to metastasis instead of primary tumors [1]. Metastatic disease represents a major clinical challenge in the treatment of cancer. Melanoma is the deadliest form of skin cancer due to its high likelihood of producing metastatic disease. Gain of function mutations in oncogene BRAF has been observed in approximately 60% of melanoma tumors. Mutational activation of BRAF results in constitutive activation of downstream molecules such as MEK and ERK leading to cell proliferation, invasion, and metastasis [2, 3]. BRAF also cooperates with the PI3K/AKT/mTOR (PI3K) signaling pathway, enhancing melanoma cell invasion and metastasis [4, 5].

To acquire invasive capabilities, primary tumor cells undergo several morphological and molecular transformations collectively known as the epithelial-mesenchymal transition (EMT). During the process of EMT, tumor cells lose their epithelial characteristics and acquire mesenchymal characteristics to become invasive [6, 7]. These morphological and molecular changes are regulated by a set of pleiotropically acting transcription factors (Snail1, Twist1, Slug and ZEB1) that are frequently expressed during EMT, leading to invasion, dissemination, and metastasis [8, 9]. Loss of E-cadherin and gain of N-cadherin is considered a hallmark of melanoma metastasis [10]. Upregulated expression of these EMT-inducing transcription factors has been associated with poor clinical prognosis [9, 11]. During metastatic melanoma progression, increased Twist1 and ZEB1 expression has been strongly correlated with a loss of E-cadherin expression. In addition to Twist1/ZEB1, Snail1 and Slug transcription factors also repress E-cadherin expression, promote N-cadherin expression, enhance MMPs expression, and increase cell survival. Furthermore, during melanoma progression transcriptional activity of ZEB1 is increased by Snail1 and Slug [12].

During melanoma progression, EMT-inducing transcription factors (Snail1, Twist1, ZEB1 and Slug) are regulated by BRAF/MEK/ERK (MAPK) and PI3K signaling [2, 11]. Activation of these EMT regulators also contributes to development of resistance to chemotherapy [8]. Since both the MAPK and PI3K pathways induce EMT and enhance cell invasion and metastasis, targeting these pathways simultaneously is a promising strategy. Sorafenib is a potent inhibitor of RAF (BRAF and CRAF) [13]. Studies have demonstrated that sorafenib induces apoptosis in cancer cells by targeting PDGFR, FGFR, c-KIT, MET, MAPK and other signaling pathways. Sorafenib also inhibits cell migration and invasion through suppression of EMT, c-MET, and MAPK pathways in different cancer cells [14, 15]. Sorafenib also inhibits *in vivo* tumor growth of different cancers implanted in nude mice by inhibiting VEGFR and angiogenesis [16, 17]. Phase II clinical studies have revealed that sorafenib is not effective as a monotherapy in patients with metastatic melanoma [16, 17].

Phytochemicals offer promising options for the management of melanoma since they can be used in combination with lower doses of existing chemotherapeutic drugs. Earlier, we demonstrated that fisetin, a naturally occurring flavonoid present in fruits and vegetables possess anti-inflammatory, anti-proliferative, pro-apoptotic and anti-tumorigenic activities against different cancers [18–23]. Treatment of human melanoma cells with fisetin decreased melanoma cell invasion and EMT progression [19]. In addition, fisetin inhibited melanoma cell proliferation and tumor growth by downregulating the PI3K/AKT/mTOR signaling pathway [24]. In the present study, we evaluated the effect of fisetin (which targets PI3K signaling) in combination with sorafenib, a multi-kinase inhibitor of mutant and wild-type BRAF and CRAF kinases, on melanoma cell invasion and metastasis. We found that a combination of fisetin and sorafenib inhibited cell migration and invasion, while abrogating EMT progression and metastasis more effectively than individual agents by modulating expression of EMT marker proteins and reducing expression of EMT-inducing transcription factors.

## RESULTS

### Combination of fisetin and sorafenib effectively inhibited migration and invasion of BRAF-mutated melanoma cells

**Figure 1:**
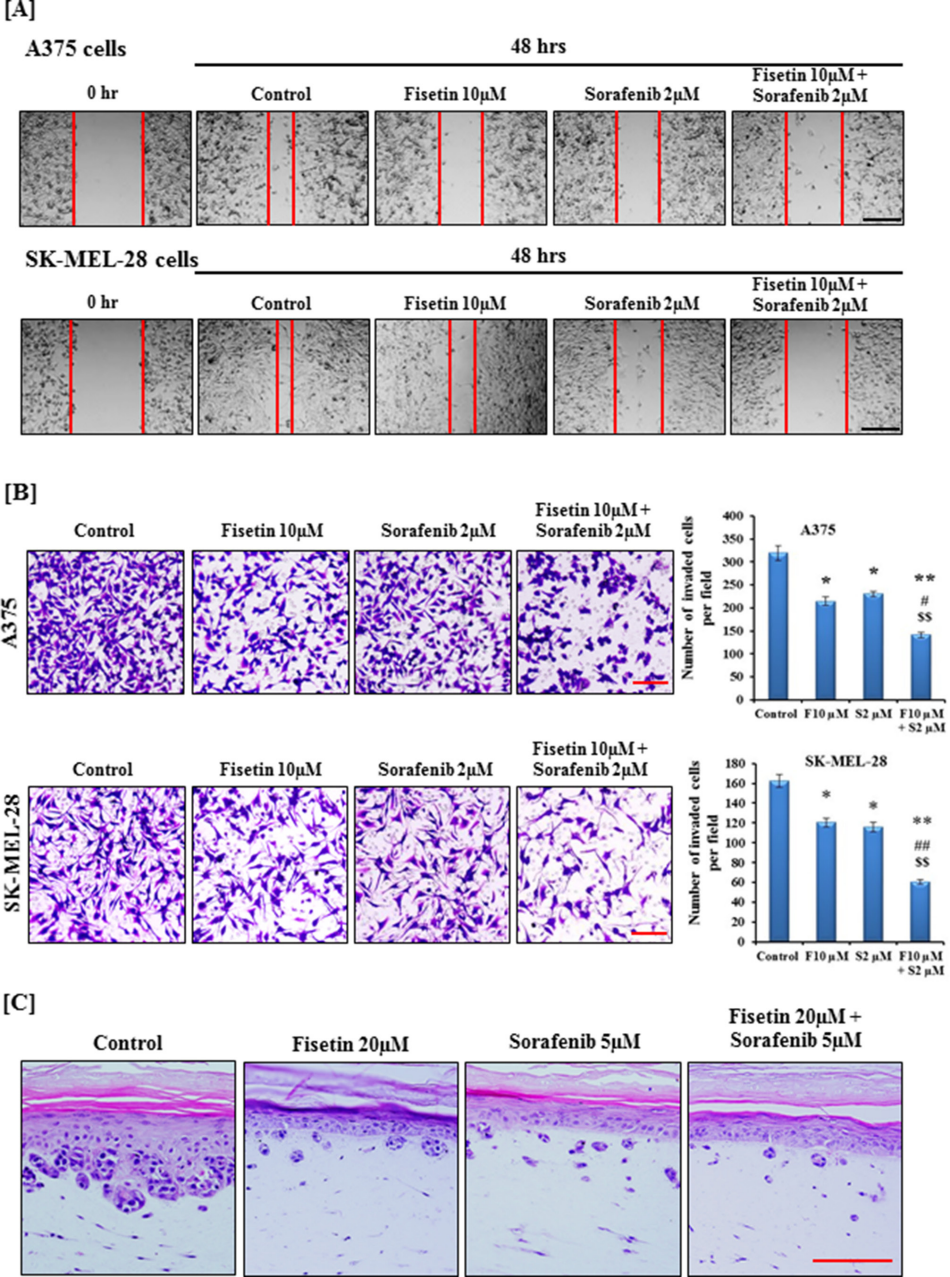
Effect of fisetin, sorafenib and their combination on migration and invasion of BRAF-mutated melanoma cells [**A**] After scratching, BRAF-mutated A375 and SK-MEL-28 cells were treated with 10 μM fisetin, 2 μM sorafenib and a combination of 10 μM fisetin and 2 μM sorafenib for 48 hrs. Images were acquired at 0 and 48 hrs under the microscope. The red lines define the areas lacking migratory cells. A representative picture from three independent experiments is shown here. Bar = 50 μm [**B**] BRAF-mutated A375 and SK-MEL-28 cells were incubated with 10 μM fisetin, 2 μM sorafenib and a combination of 10 μM fisetin and 2 μM sorafenib in a serum-reduced medium for 24 hrs in the upper chamber of a Boyden chamber while the lower chamber separated with matrigel coated polycarbonate membrane was filled with a medium supplemented with 10% FBS. After incubation, cells were fixed and stained with crystal violet and imaged under a microscope. A representative picture from three independent experiments is shown here. Bar = 5 μm. Average numbers of invaded cells were determined after counting four randomly selected microscopic fields on the membrane. *P < 0.05, **P < 0.01 significant difference versus control group. ^#^*P* < 0.05, ^##^*P* < 0.01 significant difference versus fisetin. ^$^*P* < 0.05, ^$$^*P* < 0.01 significant difference versus sorafenib. [**C**] The three-dimensional human melanoma skin equivalents containing A375 cells were treated with 20 μM fisetin, 5 μM sorafenib and a combination of 20 μM fisetin and 5 μM sorafenib for 7 days. After treatment, skin samples were collected and H&E was performed on 5 μm thin sections. A representative picture from three independent experiments is shown. Bar = 50 μm.

In order to invade and metastasize to internal organs, active migration of tumor cells is an essential step [25]. Therefore, we determined the migratory ability of BRAF-mutated A375 and SK-MEL-28 melanoma cells treated with fisetin, sorafenib and their combination at relatively non-toxic doses. Pictures were taken at 0 hr and 48 hrs after treatment as shown in Figure 1A. Treatment of A375 and SK-MEL-28 cells with fisetin (10 μM) or sorafenib (2 μM) for 48 hrs demonstrated that both fisetin and sorafenib inhibited cell migration compared to their respective control groups. Combination treatment was more effective in inhibiting cell migration compared to single agents (Figure 1A). Tumor cell dissemination starts with invasion of the basement membranes, followed by surrounding tissue, intravasation into blood vessels, extravasation at different organ sites, and finally colonization [6, 25]. Chemotaxis, which is mediated through various growth factors and their receptors, is considered as a crucial step during tumor cell dissemination [7, 8]. Therefore, we next determined the effect of fisetin, sorafenib and their combination on invasion of BRAF-mutated A375 and SK-MEL-28 melanoma cells by employing Boyden chambers in which cells were separated by matrigel coated membranes into two chambers containing different concentrations of growth factors. Assessment of density and number of invaded cells on the membrane clearly demonstrated that fisetin and sorafenib significantly inhibited melanoma cell invasion at 10 μM and 2 μM respectively after 24 hrs. Based on the number of invaded cells, fisetin (10 μM) inhibited invasion of A375 cells by 32.60% (*p* < 0.05) and sorafenib (2 μM) by 27.58% (*p* < 0.05) as compared to control. Combination treatment was more effective in reducing A375 cell invasion with a 55.79% (*p* < 0.01) reduction when compared with control. Moreover, the percentage of invaded cells was significantly lower in combination treatment compared to fisetin or sorafenib alone (Figure 1B). Similarly, in SK-MEL-28 cells combination treatment was more effective in reducing invasion (62.57%; *p* < 0.01) than fisetin (26.38%; *p* < 0.05) or sorafenib (28.83%; *p* < 0.05) treated cells (Figure 1B). The anti-invasive effects of a combination of these agents was significantly higher (*p* < 0.01) than with fisetin (10 μM) or sorafenib (2 μM) alone. Results of the Boyden chamber invasion assay clearly demonstrated that fisetin potentiated the anti-invasive potential of sorafenib in BRAF-mutated melanoma cells. The anti-invasive potential of the combination was further validated in three-dimensional human melanoma skin raft culture A375 cells admixed with normal human keratinocytes that were embedded onto a collagen-constricted fibroblast matrix. Development of vertical invasive nodes at the epidermal-dermal junction in raft cultures treated with 20 μM fisetin and 5 μM sorafenib for 7 days was reduced as compared to control. Moreover, when raft cultures were treated with a combination of fisetin and sorafenib, the anti-invasive effects were greatly enhanced compared to single agents (Figure 1C).

### Combination of fisetin and sorafenib effectively modulated EMT marker proteins and decreased expression of EMT-inducing transcription factors in BRAF-mutated melanoma cells

**Figure 2:**
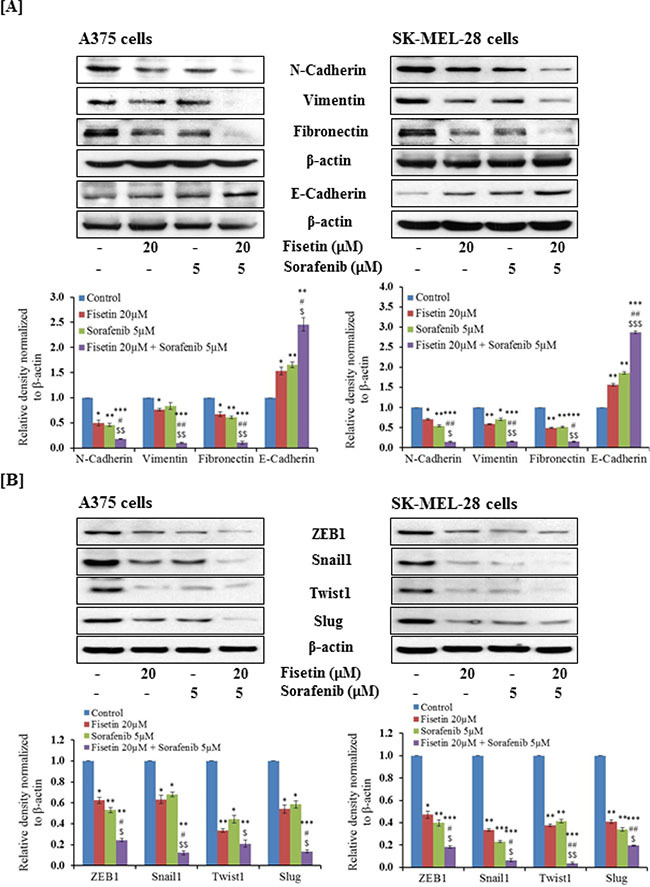
Effect of fisetin, sorafenib and their combination on protein expression of EMT-markers and EMT-inducers in BRAF-mutated melanoma cells BRAF-mutated A375 and SK-MEL-28 melanoma cells were treated with fisetin (20 μM), Sorafenib (5 μM) and a combination these agents (fisetin 20 μM + sorafenib 5 μM) for 48 hrs. 40–50 μg protein from total cell lysates was resolved over 12% Tris-glycine gels and transferred onto a PVDF membrane followed by incubation with primary antibodies of [**A**] EMT marker proteins, [**B**] EMT-inducer proteins followed by chemiluminescence detection. After stripping, membranes were analyzed for β-actin to confirm equal loading of protein. The immunoblots shown here are from a representative experiment repeated three times with similar results.

During metastasis, highly proliferative and relatively non-invasive cancer cells are transformed into highly invasive mesenchymal cells via EMT [7, 8]. Downregulation of epithelial protein such as E-cadherin and upregulation of mesenchymal proteins such as N-cadherin, vimentin and fibronectin are considered as hallmarks of EMT progression [10, 11]. Activated MAPK and PI3K signaling pathways are known to enhance EMT progression. Recently, we showed that fisetin inhibited the expression of PI3K and phosphorylation of AKT and mTOR in melanoma cell lines and xenograft tumors [24]. We determined the effects of fisetin (which targets PI3K signaling), sorafenib (an RAF inhibitor) and their combination on EMT marker proteins in BRAF-mutated melanoma cells. Western blot analysis demonstrated that fisetin and sorafenib treatment reduced the expression of mesenchymal proteins (N-cadherin, vimentin and fibronectin) and increased the expression of E-cadherin, an epithelial marker protein. Combination treatment (fisetin and sorafenib) was more effective in decreasing the expression of mesenchymal proteins as compared to individual agents (Figure 2A). In addition, combination treatment was more effective in enhancing expression of the epidermal marker protein E-cadherin when compared to individual agents (Figure 2A). During EMT progression, transcriptional repression of E-cadherin is regulated by a subset of E-box binding transcriptional factors including Snail1, Twist1, Slug and ZEB1 [9, 11]. These transcription factors are potent enhancers of melanoma cell migration and invasion and mediate resistance to apoptosis. Expression of these EMT-inducers is regulated by MAPK and PI3K signaling, therefore, the effect of fisetin, sorafenib and their combination on the expression of these EMT-inducing transcription factors in BRAF-mutated melanoma cells was also determined. As shown in Figure 2B, fisetin and sorafenib reduced the expression of Snail1, Twist1, Slug and ZEB1 in BRAF-mutated melanoma cells. Combination treatment was more effective in reducing expression of Snail1, Twist1, Slug and ZEB1 proteins when compared to individual agents (Figure 2B).

### Combination of fisetin and sorafenib effectively modulated the expression of EMT marker proteins in xenograft tumors implanted with BRAF-mutated melanoma cells

**Figure 3:**
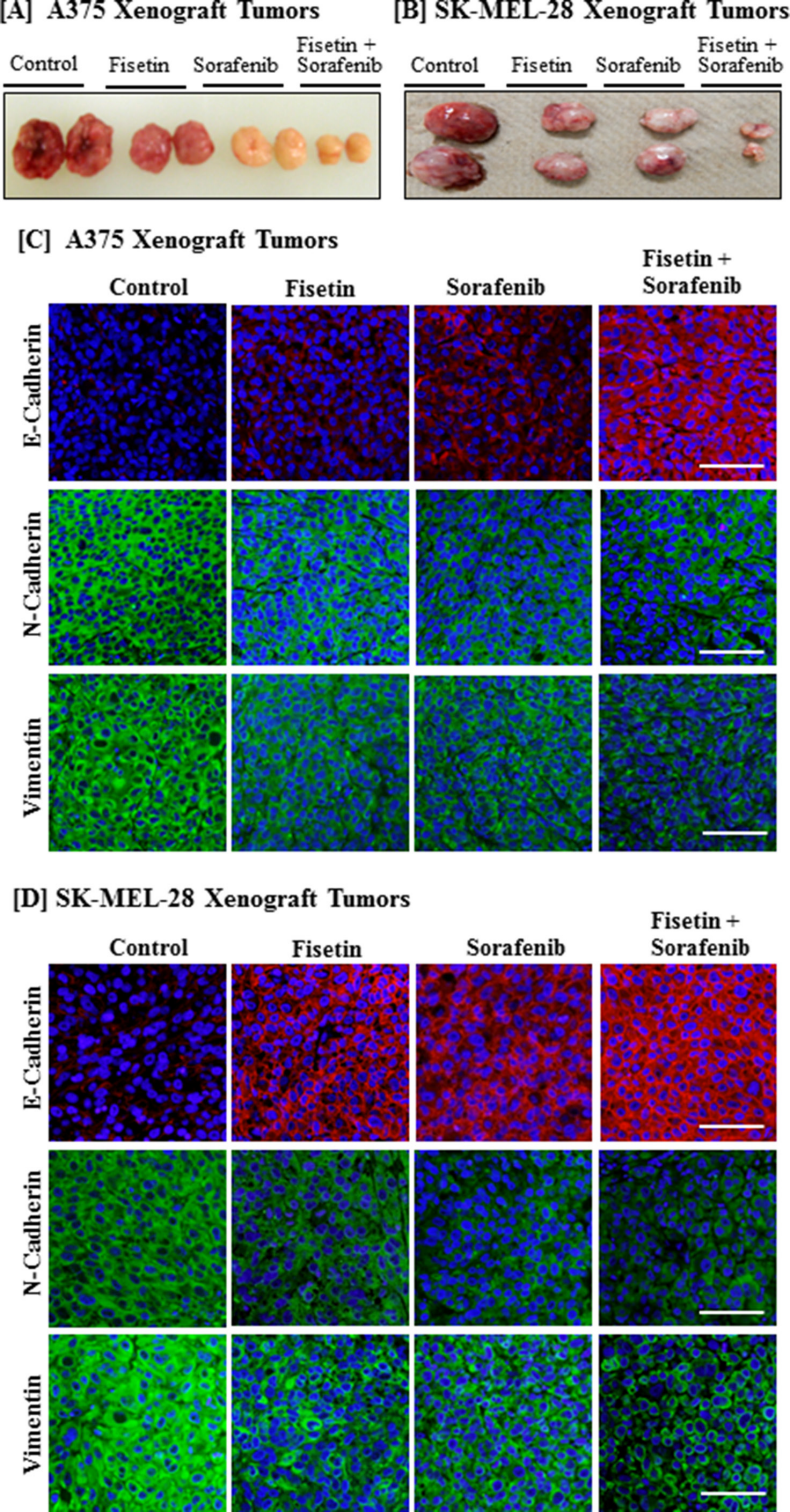
Effect of fisetin, sorafenib and their combination on expression of EMT marker proteins in BRAF-mutated melanoma xenograft tumors Athymic nude mice implanted with A375 or SK-MEL-28 cells were treated with fisetin 45 mg/kg p.o., sorafenib 45 mg/kg p.o., and their combination (fisetin 45 mg/kg + sorafenib 45 mg/kg) p.o. three times/week. All the mice were euthanized when tumor size reached 1200 mm^3^ in control group. [**A**] & [**B**] Images show representative tumors excised from the mice. [**C**] & [**D**] Tumor tissues were collected in 10% formalin and blocks were prepared in paraffin and immunostaining of EMT marker proteins (N-Cadherin, Vimentin and E-Cadherin) was performed. Images shown here are representative pictures taken from three independent tumor samples. Bar = 20 μm.

We have shown that athymic nude mice implanted with BRAF-mutated A375 and SK-MEL-28 melanoma cells treated with fisetin (45 mg/kg, body weight) and sorafenib (45 mg/kg, body weight) in combination more effectively reduced tumor growth compared to the individual agents alone [24]. The dosing for these agents was safe and showed no apparent signs of toxicity in these mice when the agents were used alone and in combination. A representative picture of these tumors is shown in Figure 3A and 3B. When sections of these archival tumor samples were stained with EMT-related antibodies, we found that fisetin and sorafenib enhanced the expression of E-cadherin (an epithelial marker protein) in xenograft tumors implanted with A375 and SK-MEL-28 melanoma cells. The combination treatment was more effective in inducing the expression of E-cadherin than individual agents (Figure 3C and 3D). Furthermore, the combination treatment was more effective than individual agents in reducing expression of N-cadherin, vimentin and fibronectin mesenchymal marker proteins in melanoma xenografts implanted with A375 and SK-MEL-28 cells (Figure 3C and 3D).

### Combination of fisetin and sorafenib resulted in greater reduction in the expression of EMT-inducing transcription factors

**Figure 4:**
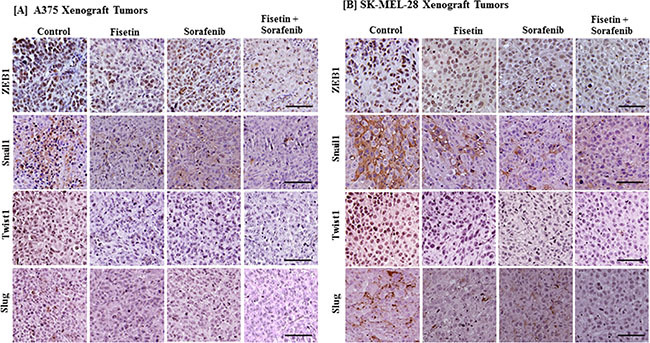
Effect of fisetin, sorafenib and their combination on expression of EMT-inducing protein in BRAF-mutated melanoma xenograft tumors Tumor sections from the [**A**] A375 or [**B**] SK-MEL-28 xenografts of control and treated mice were stained with antibodies of EMT-inducing transcription factors (ZEB1, Snail1, Twist1 and Slug). Images shown here are representative pictures taken from three independent tumor samples. Bar = 20 μm.

Since combination treatment effectively reduced melanoma cell invasion and modulated the expression of EMT marker proteins *in vitro* as well as in melanoma xenografts, we next determined the effect of combination treatment on EMT-inducing transcription factors. Studies have shown that EMT-related transcription factors such as Snail1, Twist1, Slug and ZEB1 are also regulated by MAPK and PI3K signaling. Co-targeting of MAPK and PI3K by sorafenib and fisetin resulted in greater reduction in mesenchymal marker proteins, so tumor sections were further evaluated for expression of EMT-inducing transcription factors. We found that fisetin and sorafenib treatment reduced the expression of Snail1, Twist1, Slug and ZEB1 proteins. More importantly, their expression was greatly reduced in combination treatment as compared to treatment with individual agents (Figure 4A and 4B).

### Combination of fisetin and sorafenib effectively reduced MMPs expression in xenograft tumors of BRAF-mutated melanoma cells

**Figure 5:**
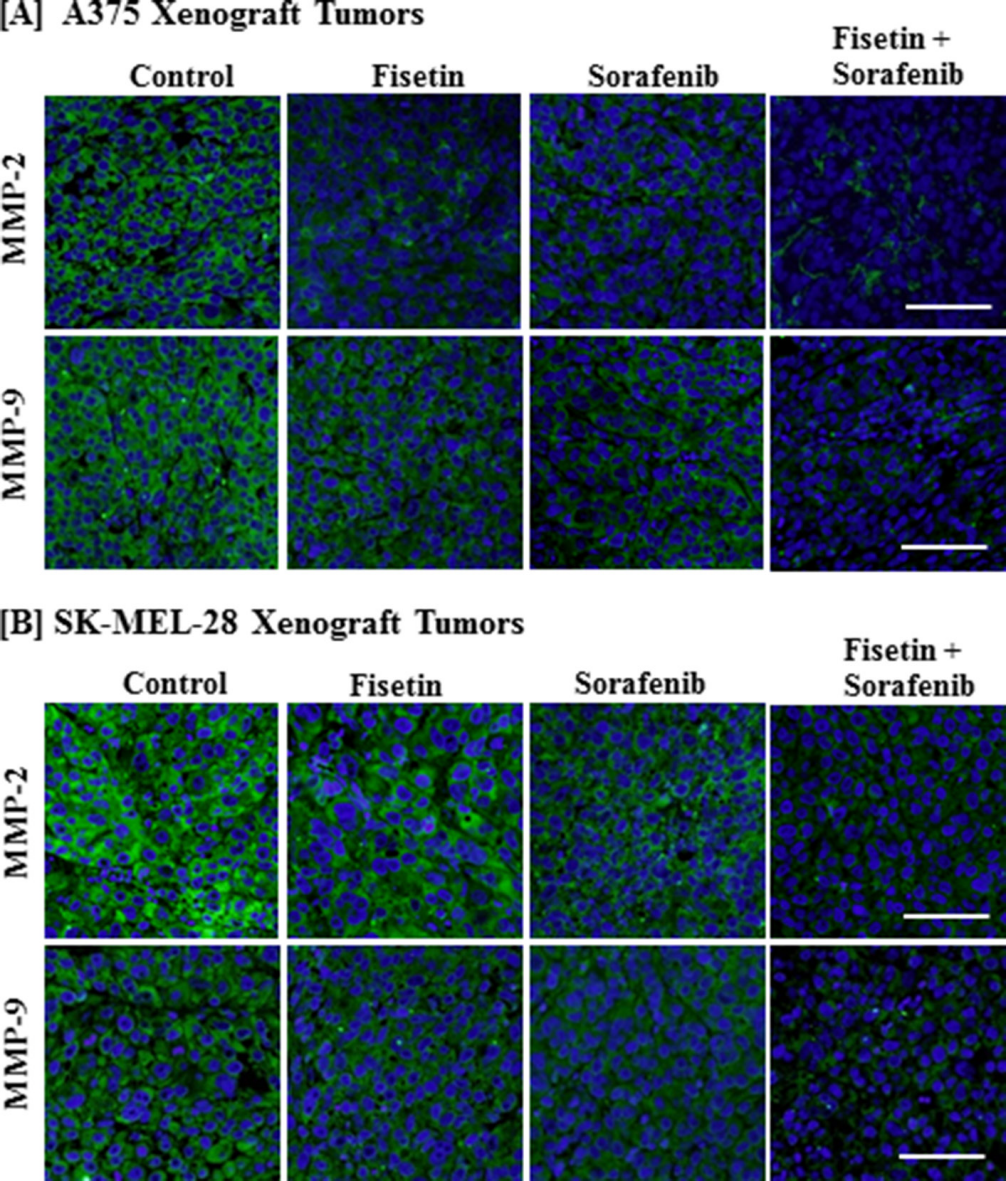
Effect of fisetin, sorafenib and their combination on MMP-2 and MMP-9 expression in BRAF-mutated melanoma xenograft tumors Tumor sections from the [**A**] A375 or [**B**] SK-MEL-28 xenografts of control and treated mice were stained with antibodies of MMP-2 and MMP-9. Images shown here are representative pictures taken from three independent tumor samples. Bar = 20 μm.

During cell migration, extracellular matrix (ECM) remodeling or degradation is an essential step [26]. MMPs are a family of proteinases that play an important role in remodeling or degradation of ECM and basement membranes. Their dysregulation leads to invasion, angiogenesis and tumor metastasis [27]. In melanoma, MMP-2 and MMP-9 overexpression has been associated with EMT, invasion, and metastasis [28, 29]. In particular, MMP-2 overexpression has been correlated with tumor progression and poor survival in melanoma patients [30, 31]. Therefore, we determined the expression of MMP-2 and MMP-9 in tumor sections. We found that fisetin and sorafenib alone were able to reduce MMP-2 and MMP-9 expression, but the combination of fisetin and sorafenib was more effective than individual agents in reducing the expression of these proteins (Figure 5A and 5B).

### Combination of fisetin and sorafenib effectively reduced lung metastasis of BRAF-mutated melanoma cells injected intravenously

**Figure 6:**
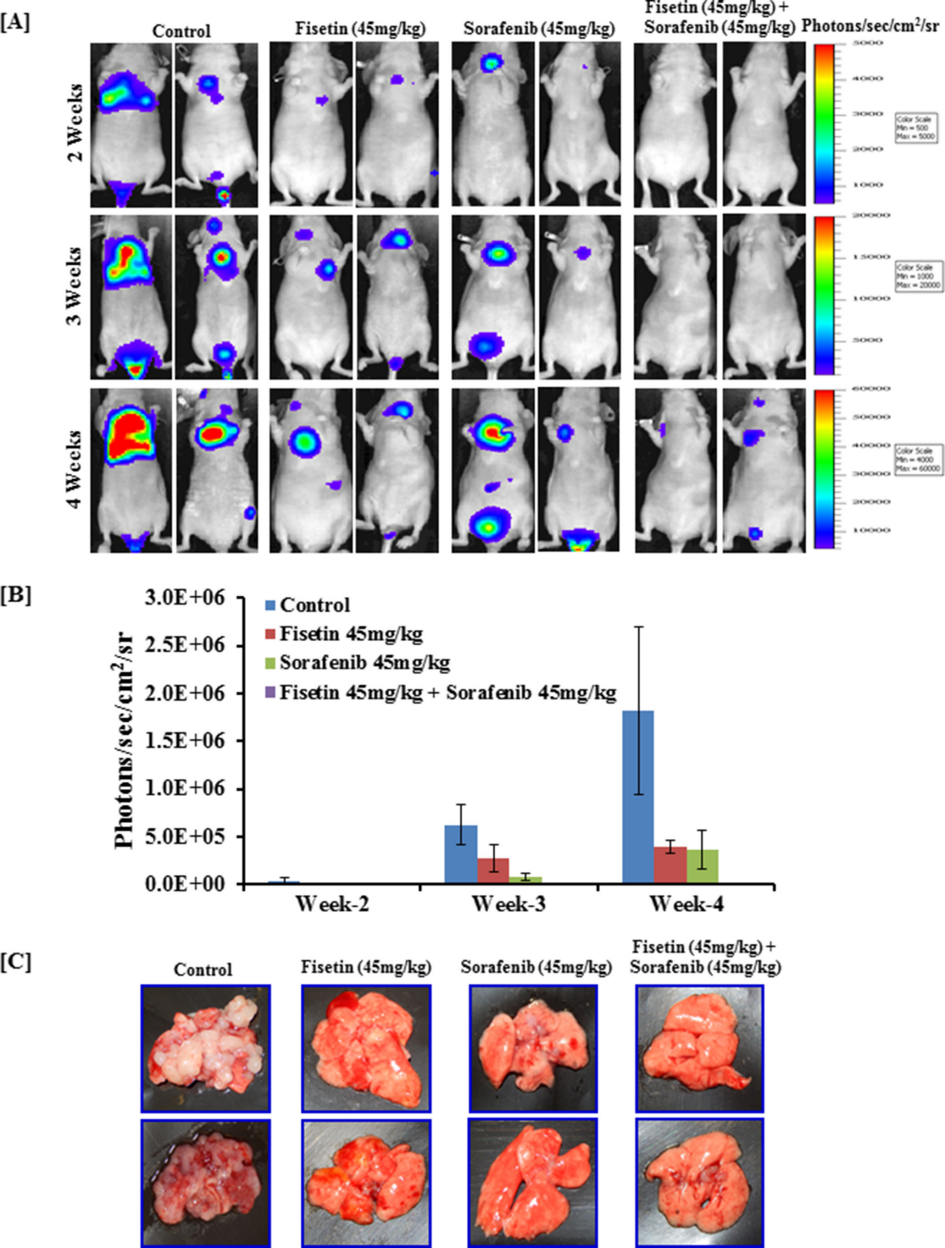
Effect of fisetin, sorafenib and their combination on lung metastasis (colonization) assay in nude mice intravenously injected with BRAF-mutated melanoma cells Athymic nude mice were intravenously injected with 2×10^6^ A375 cells in 0.2 ml PBS in the tail vein. Mice were treated with fisetin 45 mg/kg p.o., sorafenib 45 mg/kg p.o., and their combination (fisetin 45 mg/kg + sorafenib 45 mg/kg) p.o. three times/week as described in “Methods” section. [**A**] Mice (*n* = 4) were imaged to record photon emission from tumors using a cooled CCD camera apparatus (IVIS, Xenogen) after i.p. injection of 100 μl of D-luciferin (30 mg/ml) (Xenogen, Alameda, CA, USA). [**B**] Living Image software (Xenogen) was used to analyze the data. [**C**] At the end of the experiment, all mice were euthanized, lungs were removed, and images were taken by digital camera. Images shown here are representative pictures taken from three mice of the same groups.

Lungs are the most frequent site of malignant melanoma metastasis. Lung tumor formation after intravenous tail vein injection of melanoma cells is the most frequently used model to study melanoma metastasis and to evaluate the anti-metastatic potential of therapeutic agents [32]. Therefore, we utilized this experimental metastasis model to test whether fisetin, sorafenib and their combination inhibits melanoma metastasis progression. For this, athymic nude mice were intravenously injected with stably transfected CMV-luciferase-ires-puromycin. T2A.EGFP-tagged BRAF-mutated A375 human melanoma cells. The progression of lung metastasis was monitored and quantified using a non-invasive bioluminescence technique [32]. Bioluminescence data at weeks 2 to 4 revealed that after tail vein injection of melanoma cells, lung metastasis was established and progressed exponentially in number and size in the control group (Figure 6A and 6B). Whereas in the fisetin or sorafenib treated group, rate and number of lung metastases formation were slower compared to the control group. Combined treatment with fisetin and sorafenib was the most effective, as it almost halted progression of the lung metastases (Figure 6C). Bioluminescence data was further verified by necropsy at the end of the experiment at week 4. As shown in the representative images of the lungs, combination treatment more effectively inhibited formation of lung metastases when compared to the lungs of control mice. Fisetin and sorafenib alone treatment also reduced lung metastases but not to the extent that was observed in combination treatment (Figure 6C).

## DISCUSSION

Despite advancement in melanoma diagnosis along with revolutionary progress in targeted therapies, treatment of malignant melanoma after distant organ metastasis remains challenging [3, 33]. Melanoma incidence is increasing at an alarming rate. According to a recent estimate, 73,870 people will be diagnosed with cutaneous melanoma and 9,940 people will die from this disease in the USA in 2015 [34]. Caucasian populations are approximately 10-fold more likely to develop melanoma than deeply pigmented skin populations [34]. The 5-year survival rate for localized melanoma patients is over 90%; however, if the tumor metastasizes to distal organs the survival rate drops to ∼10% [3]. Relapse and aggressive progression of melanoma occurs in most of the patients after 5–7 months of treatment with BRAF inhibitors due to re-activation of MAPK and/or cooperation of PI3K and other alternative signaling pathways [4, 5, 35]. In the present study we investigated sorafenib, a RAF inhibitor in combination with fisetin on cell invasion, EMT and lung metastasis of BRAF-mutated melanoma cells. Our results demonstrate that simultaneous targeting of MAPK by sorafenib and PI3K signaling by fisetin in melanoma cells more effectively inhibited cell migration and invasion when compared to treatment with individual agents.

Transformation of a non-motile polarized epithelial phenotype to a motile and invasive mesenchymal phenotype is frequently associated with poor prognosis and high risk of metastasis [9, 11]. The cadherin switch during EMT in melanoma is considered a hallmark of invasiveness and metastatic progression [10]. Furthermore, oncogenic BRAF signaling has been shown to suppress E-cadherin expression and enhance melanoma cell invasion by inducing T-Box3 transcriptional repressor of E-cadherin expression [36]. In addition, the PI3K/PTEN pathway also plays an important role in the cadherin switch by suppressing E-cadherin expression and enhancing N-cadherin expression [35]. It has been shown that sorafenib inhibits cell migration and invasion of various cancer cells by reducing EMT [14, 15]. Our previous study demonstrated that fisetin inhibits melanoma cell invasion by reducing expression of mesenchymal marker proteins and upregulating expression of epithelial marker proteins [19]. In this investigation when a low dose of fisetin and sorafenib were combined, combination treatment exhibited greater reduction in mesenchymal marker protein expression (N-cadherin, vimentin and fibronectin) and potentiated upregulation of epithelial marker protein expression (E-cadherin) in BRAF mutated melanoma cells in both *in vitro* and *in vivo* xenograft tumors.

The process of EMT is driven by several transcription factors such as Snail1, Twist1, Slug and ZEB1. Aberrant expression of these transcription factors in melanoma has been associated with progression of metastasis and poor prognosis [9, 11, 37]. The transcription factor Snail1 has been associated with EMT induction, enhanced migratory potential, and metastasis of melanoma cells via transcriptional repression of E-cadherin expression [38, 39]. Oncogenic BRAF in melanoma cells induces EMT through upregulation of Snail1 expression [40]. Moreover, cooperation of PI3K/AKT signaling also upregulates Snail1 expression leading to PTEN downregulation in melanoma cells [2, 41]. A growing body of evidence has shown that sorafenib inhibits EMT, Snail1 expression, and E-cadherin to N-cadherin switching in various tumors [14, 42, 43]. In the present study, analysis of sorafenib treated BRAF-mutated melanoma cells and xenograft tumor tissues showed reduced protein expression of mesenchymal marker proteins (N-cadherin, vimentin and fibronectin). The protein expression of E-cadherin (a marker of epithelial characteristics) was increased in sorafenib treated melanoma cells as well as in xenograft tumor tissues. Moreover, sorafenib treatment reduced the protein expression of EMT-related transcription factors Snail1, Twist1, Slug and ZEB1 in these samples. We found that fisetin also inhibited expression of Snail1 in BRAF-mutated melanoma cells *in vitro* as well as in xenograft tumors. Moreover, a combination of fisetin and sorafenib effectively reduced the Snail1 expression, resulting in a marked increase in E-cadherin expression in BRAF-mutated melanoma cells, suggesting that fisetin potentiated the EMT-inhibitory potential of sorafenib. E-cadherin expression is down regulated by Twist1, ZEB1 and Slug transcription factors [9, 11]. Twist1 is crucial for EMT and melanoma cell migration. Enhanced expression of Twist1 has been associated with poor prognosis and metastasis. Twist1 can suppress E-cadherin expression and promote upregulation of N-cadherin and fibronectin independently of Snail1 by ERK1/2 signaling [44, 45]. Furthermore, activated PI3K signaling overcomes BRAF mediated senescence and potentiates environmental escape by inducing EMT in melanoma cells [46]. In the present investigation, when sorafenib was combined with fisetin, fisetin potentiated the EMT inhibitory effect of sorafenib and effectively down regulated the expression of Twist1 transcription factor in BRAF-mutated melanoma cells *in vitro* as well as in xenograft tumors. ZEB1, another oncogenic transcription factor similar to Twist1, also cooperates with BRAF signaling and is also implicated in E-cadherin suppression, EMT induction, and metastasis [9, 11, 47]. Expression of ZEB1 is enhanced by another EMT-inducing transcription factor, Slug. Slug is a direct repressor of E-Cadherin expression through E-box interaction and promotes tumor cell invasion and metastasis [48]. In melanoma cells, Slug also acts as a direct transcriptional activator of E-box at the ZEB1 promoter, and cooperatively repressed E-cadherin expression resulting in increased migration and metastasis of melanoma cells [12]. Slug induced upregulation of cell invasion is AKT dependent [49]. In this study, when fisetin was combined with sorafenib, the combination produced greater inhibition of Slug and ZEB1 protein expression. This demonstrate that fisetin further potentiated the inhibitory effects of sorafenib against EMT-related transcription factors both *in vitro* and *in vivo*.

Expression of MMP-2 and MMP-9 has also been shown to promote cell invasion and metastasis of melanoma. Overexpression of MMP-2 has been implicated in melanoma progression and survival. In addition to degradation of the extracellular matrix, MMPs expression also promotes EMT progression [28]. Furthermore, MMPs play a critical role in the development of malignant angiogenesis. A growing body of evidence has demonstrated that MMPs are implicated in the release of the proangiogenic factor VEGF [50–52]. It has been demonstrated that sorafenib inhibits *in vivo* tumor growth by inhibiting VEGFR signaling and angiogenesis [16, 17]. This fact has also been validated by our previously published study in which we found that sorafenib inhibited protein expression of angiogenesis markers VEGF and CD31 in melanoma xenograft tumor tissues. Moreover, anti-angiogenic effect of sorafenib was greatly enhanced when combined with fisetin [24]. It has also been shown that EMT transcription factors activate MMPs expression. This then leads to upregulated MMPs, which further activates EMT transcription factors in a feed-forward loop [53]. Treatment with single agents or a combination of fisetin and sorafenib both effectively modulated markers of EMT and reduced transcription factors related to EMT progression and expression of MMP-2 and MMP-9. In combination treatment however, more significant reduction in MMP-2 and MMP- 9 levels demonstrated that the expected inhibitory effect of sorafenib was augmented due to combination with fisetin.

More importantly, the *in vitro* anti-invasive effects of fisetin and sorafenib against BRAF-mutated melanoma were translated under *in vivo* conditions. Simultaneous targeting of MAPK and PI3K, two parallel signaling pathways involved in EMT induction, invasion and metastasis, resulted in greater inhibition in lung metastases of melanoma cells by sorafenib and fisetin compared to individual agents alone. These data suggest that fisetin further potentiated the anti-metastatic effect of sorafenib.

Our findings demonstrated that fisetin inhibits melanoma cell invasion, EMT progression and metastasis of BRAF-mutated melanoma cells by modulating the expression of EMT marker proteins and reducing transcription factors related to EMT. Furthermore, our findings demonstrated fisetin's ability to potentiate the anti-invasive and anti-metastatic effects of sorafenib against BRAF-mutated melanoma cells under *in vitro* as well as *in vivo* conditions. Based on our data we suggest that the use of the dietary compound fisetin, along with other such cell-signaling targeted therapies may be a promising strategy for the management of metastatic melanoma.

## MATERIALS AND METHODS

### Reagents and antibodies

Fisetin (purity ≥ 98%) and β-actin mouse monoclonal antibody were obtained from Sigma-Aldrich (St. Louis, MO, USA). Sorafenib Tosylat N Mikron (BAY 43– 90006) was obtained as a gift from Bayer Health Care (Bayer Pharma AG, Berlin, Germany). Antibodies for E-Cadherin, N-Cadherin, Vimentin, Snail1, Slug, Twist1, ZEB1, Fibronectin, MMP-2 and MMP-9 were obtained from Cell Signaling Technology (Danvers, MA, USA). Goat anti-rabbit, rabbit anti-goat and rabbit anti-mouse antibodies conjugated with horseradish peroxidase were purchased from Millipore Corporation (Billerica, MA, USA). Fluorchrome Alexa Fluor 488/594 conjugated goat anti-rabbit, rabbit anti-goat, and rabbit anti-mouse were purchased from Life Technologies (Grand Island, NY, USA). Three-dimensional human melanoma skin equivalents of A375 cells (MLNM-FT-A375) were obtained from MatTek Corporation (Ashland, MA, USA).

### Cell culture

Human malignant melanoma cells (A375) were obtained from American Type Culture Collection (Manassas, VA, USA) and cultured in DMEM medium. SK-MEL-28 cells were obtained from Alan Houghton, Sloan-Kettering Institute for Cancer Research (New York, NY, USA) and cultured in PRMI-1640 medium. DMEM and RPMI-1640 medium were supplemented with 10% heat inactivated fetal bovine serum and 100 mg/ml penicillin-streptomycin solution. Cells were maintained at 37°C and 95% humidified incubator with 5% CO_2_.

### Migration assay

To test the anti-migratory potential of fisetin, sorafenib and their combination, melanoma cells were seeded in 35 mm cell culture dishes and incubated overnight. The monolayer of the cells was scratched with a sterile 10 μl pipette tip. Cells were then washed twice with PBS to remove detached cells from the plates. Cells were then incubated in a medium containing fisetin (10 μM), Sorafenib (2 μM) and a combination these agents (fisetin 10 μM + sorafenib 2 μM) for 48 hrs. Fisetin and sorafenib were dissolved in DMSO and final concentration of DMSO in each treatment was <0.1% (v/v). Cell migration was determined by imaging the scratch under phase-contrast microscope.

### Invasion assay

To evaluate the anti-invasive potential of fisetin, sorafenib and their combination, we performed transmembrane invasion of A375 and SK-MEL-28 melanoma cells using a Boyden chamber assay. In this assay, two chambers were separated by matrigel coated 8 μm porous membranes. Approximately 3×10^4^ melanoma cells were placed in the upper chamber of the Boyden chamber containing 10 μM fisetin, 2 μM sorafenib or combination of 10 μM fisetin and 2 μM sorafenib in a serum reduced medium (containing 1% FBS). The lower chamber was filled with 110 μl medium supplemented with 10% FBS. Chambers were incubated at 37°C in a 95% humidified incubator with 5% CO_2_. After 24 hrs of incubation, cells from the upper surface of the membranes were removed with gentle swabbing. Invaded cells on the lower surface of the membranes were fixed with chilled methanol and stained with crystal violet (0.5% crystal violet in 25% methanol). The membranes were then washed with distilled water three times and mounted onto glass slides and were examined microscopically.

### Treatment of three-dimensional human melanoma skin equivalents

The three-dimensional human melanoma skin equivalents of A375 cells (MLNM-FT-A375) admixed with normal human keratinocytes at a ratio of 1:5 that were embedded onto a collagen-constricted fibroblast matrix were purchased from MatTek Corporation (MatTek, Ashland, MA). Inserts were cultured in MLNM-FT-MM medium at 37°C and 5% CO_2_ in a humidified chamber to form highly differentiated human melanoma three-dimensional skin equivalents. The three-dimensional skin equivalents were maintained at air-liquid interface with the lower dermal side of the tissue exposed to media and the upper epidermal stratum corneum exposed to air. These skin raft cultures were treated with 20 μM fisetin, 5 μM sorafenib or combination of 20 μM fisetin and 5 μM sorafenib in 5 ml MLNM-FT-MM medium for 7 days. Treatment was replaced every alternate day with freshly prepared treatment. At the end of the treatment duration, skin from the inserts were collected, fixed in 10% neutralized formalin and embedded in paraffin. Hematoxylin and eosin staining was performed on 5 μm thin sections after deparaffinization in xylene and rehydration through graded ethanol. The skin sections were examined microscopically for development of nodes at the epidermal-dermal junction and vertical invasion of metastatic melanoma A375 cells into the dermis.

### *In vivo* tumor growth

Female athymic nude mice of five weeks age were subcutaneously transplanted with A375 or SK-MEL-28 cells (2.5 × 10^6^ A375 cells in 50 μl DMEM + 50 μl matrigel or 5 × 10^6^ SK-MEL-28 cells in 50 μl RPMI + 50 μl matrigel) in each flank and divided into four groups with six mice in each group. Mice of groups II, III and IV were treated orally three times a week with fisetin 45 mg/kg, sorafenib 45 mg/kg, and with their combination (fisetin 45 mg/kg + sorafenib 45 mg/kg) suspended in 5% cremophor + 2% ethanol in water respectively. Mice of the first group received an equal volume of the vehicle orally, and served as control. Tumor size was recorded and the animals were sacrificed when tumors reached a volume of ∼1200 mm^3^ in the control group as described earlier [24].

### Preparation of cell lysates

A375 and SK-MEL-28 melanoma cells were treated with 20 μM fisetin, 5 μM sorafenib or combination of 20 μM fisetin and 5 μM sorafenib for 48 hrs. After incubation, the medium was aspirated and the cells were washed with PBS (10mmol/l, pH 7.45). The cells were then incubated in an ice cold lysis buffer containing 10 mM HEPES (pH 7.9), 100 mM KCl, 10 mM EDTA, 20 mM EGTA, 100 mM DTT, 20 mM PMSF, 0.5% NP-40 with a freshly added protease inhibitor cocktail containing leupeptin, aprotinin and benzamidine for 20 min. Lysates were collected by scraping and passed through a 21.5-G needle to break up the cell aggregates. The lysates were cleared by centrifugation at 14,000 g for 10 min at 4°C, and the supernatant was collected, aliquoted, and used on the day of preparation or immediately stored at −80°C for future use.

### Western blot analysis

To perform Western blot analysis of cell lysates prepared after 48 hrs of treatment with 20 μM fisetin, 5 μM sorafenib or a combination of 20 μM fisetin and 5 μM sorafenib, 40–50 μg protein was resolved over 8–12% Tris–glycine gels and transferred onto polyvinylidene fluoride (PVDF) membranes. The membranes were incubated with a blocking buffer containing 5% non-fat dry milk/0.1% Tween-20 in Tris-Buffered Saline (TBS), pH 7.6 for 1 hr at room temperature. Membranes were incubated over night at 4°C with appropriate primary antibodies in a blocking buffer. After washing with TBST for 5 mins, membranes were incubated with anti-mouse or anti-rabbit secondary antibody horse-radish peroxidase conjugate for 1 hr at room temperature. Membranes were then washed with TBST washing buffer and detected by chemiluminescence using a Pierce ECL Western Blotting Substrate (Thermo Scientific, Rockford, IL) and autoradiography using a HyBlot CL Autoradiography Film obtained from Densville Scientific Inc., (Metuchen, NJ). The ImajeJ scientific software program (http://rsbweb.nih.gov/ij/index.html) was used to measure band density.

### Immunohistochemistry and immunofluorescence staining

After harvesting, the melanoma xenograft tumors were fixed in formalin and embedded in paraffin. Five micrometer sections of these tumors were deparafinized in xylene and then hydrated through a series of xylene and ethanol washes. Sections were incubated in a citrate buffer (pH 6.0) for 30 min at 95°C for antigen retrieval. Endogenous peroxidase activity was quenched by incubating sections with 3% H_2_O_2_ for 20 min at room temperature. After blocking, sections were incubated overnight with appropriate dilatation of primary antibodies followed by incubation with a secondary antibody either conjugated with HRP or fluorochrome. Sections were washed three times with PBS for incubation with diaminobenzidene peroxidase substrate (DAB) or Vectashield mounting media containing DAPI.

### Generation of EGFP/luciferase reporter A375 melanoma cells

To facilitate detection and quantification of experimental metastasis *in vivo*, A375 cells were transduced with a vesicular stomatitis virus G envelope (VSV-G) pseudotyped lentiviral vector for constitutive expression of both firefly luciferase and enhanced green fluorescence protein (EGFP). The vector (designated K2947) comprised the mouse CMV promoter, followed by fire fly luciferase, the encephalomyocarditis internal ribosomal entry site (IRES), a puromycin resistance gene (puro) and the enhanced green fluorescence protein (EGFP), wherein puro and EGFP were fused in-frame at their 3′ and 5′ ends, respectively, with the “self-cleaving” T2A peptide-coding sequence (CMV-luciferase-IRES-puro.T2A.EGFP) [54]. The lentiviral vector, the packaging construct, and the VSV-G plasmid DNAs were cotransfected into 293T human embryo kidney cells to create infectious, replication defective, lentiviral vector-containing particles as described earlier [55]. A375 cells were transduced with the vector using a multiplicity of infection of approximately 5. Stable, expression-positive A375 cells were selected by supplementing the culture medium with 5 μg/ml of puromycin for five days.

### Lung metastasis (colonization) study

Athymic nude mice (6 week old) were obtained from the National Cancer Institute-Frederick National Laboratory for Cancer Research and housed in animal facility of University of Alabama at Birmingham under pathogen-free conditions with 12 hrs light/12 hrs dark schedule at 24 ± 2°C temperature, 50% ± 10% relative humidity. Lung metastasis experiments in mice were performed in accordance with the guidelines and animal protocol number (IACUC-09709) approved by the Institutional Animal Care and Use Committee (IACUC) of University of Alabama at Birmingham. Mice were fed with phytochemical free diet AIN-76 SEMI PD (Test Diet, Richmond, IN, USA) and water *ad libitum*. For *in vivo* lung metastasis studies, on the day of inoculation, mice were warmed briefly under a heat lamp to dilate their veins. Each mouse was then injected with 2 × 10^6^ stably transfected A375 cells in 0.2 ml PBS via lateral tail vein. Mice were randomly divided in to four groups (*n* = 6 each group). Twenty-four hours after cell implantation, mice of the first group received 100 μl of vehicle orally and served as control. Mice of the Group II, III and IV were treated orally with fisetin (45 mg/kg b.wt), sorafenib (45 mg/kg b.wt.), and with their combination (fisetin 45 mg/kg b.wt. + sorafenib 45 mg/kg b.wt.) suspended in 5% cremophor + 2% ethanol in water respectively, three times a week. At different time points, mice were imaged by i.p. injection of 100 μl of D-luciferin (30 mg/ml) (Xenogen, Alameda, CA, USA). Prior to imaging mice were anesthetized and then placed in a light tight chamber and a cooled CCD camera apparatus (IVIS, Xenogen) was used to detect photon emission from tumor bearing mice at different acquisition time periods. Imaging data was analyzed on Living Image software (Xenogen) by drawing the region of interest over the tumor region to obtain maximum photons per second per cm^2^ per steradian as described earlier [56]. At the end of the experiment, all mice were euthanized and lungs were removed for imaging and further analysis.

### Statistical analysis

Data are shown as mean ± SEM. Levels of significance of differences among the groups were calculated using Student's *t*-test. *p* < 0.05 was considered to be statistically significant.
